# Central Ohio Dental Association

**Published:** 1861-03

**Authors:** J. B. Beauman


### CENTRAL OHIO DENTAL ASSOCIATION.

In pursuance of a call, informally made only a few weeks since, the dental profession was very creditably represented at the office of Dr. Riley, in the city of Columbus, on the 15th of January, 1861.

The meeting was called for the purpose of consultation on matters of professional interest, and for entering into such an arrangement as in the united judgment of the members present, would contribute most efficiently towards the elevation of the profession in Central Ohio, and of establishing a more friendly interchange of professional courtesy and good feeling.

On motion, Dr. Thos. McCune, of Columbus, was called to the chair, and Dr. J. G. Hamill, of Lancaster, was chosen Secretary.

Dr. Hamill moved that the President appoint a committee of three, to report a Constitution for the direction and government of a permanent organization. Adopted.

The President appointed Drs. Harrington, Riley and Hamill said committee.

After a short delay, the committee reported the following Constitution, which, on motion of Dr. Sedgwick, was received, and the committee discharged.

On motion of Dr. Harrington, the Constitution was taken up and considered, and voted upon by articles, all of which, after some amendments, were adopted.

On motion of Dr. Beauman, the Constitution, as amended, was adopted as a whole, and the members of the profession present, with one exception, subscribed their names thereto.

### CONSTITUTION.

Article 1.—This Society shall be known by the name of the Central Ohio Dental Association.

Art. 2.—The objects of this association shall be the improvement of its members in the art and science of dentistry, and the cultivation of a more fraternal and reciprocal interchange of kindly feelings personally.

Art. 3.—The officers of this association shall consist of a President, a Vice-President, a Recording Secretary, a Corresponding Secretary, and a Treasurer.

Art. 4.—The officers of this association shall be elected by ballot annually, a majority vote only being necessary for a choice.

Art. 5.—The duties of the officers shall be the same as appropriately belong to such offices in all deliberative bodies.

Art. 6.—The membership of this association shall consist of all practicing dentists present at each meeting, who shall subscribe, or who shall have subscribed to this Constitution, and pay the assessment necessary for defraying the incidental expenses of each meeting, as herein-after provided for.

Art. 7.—This association shall be composed of two classes—acting and honorary members.

Art. 8.—The meetings of this association shall be at such time and place as a majority of the members present at each meeting may decide.

Art. 9.—A majority of the members present at any meeting shall constitute a quorum for the transaction of business.

Art. 10.—The expenses attendant upon each meeting shall be paid by an equal assessment upon each of the members present at such meeting.

Art. 11.—This constitution may be altered or amended by a vote of two-thirds of the members present at any regular meeting, notice of such contemplated change having been given at the previous meeting.

After the signing of the Constitution, the election of officers, as therein provided for, was entered upon; and after due course of balloting, the following named members were declared elected to the several offices respectively:

Dr. J. G. Hamill, *President;*

" W. W. Riley, *Vice-President;*

" J. B. Beauman, *Recording Secretary;*

" Thomas M'Cune, *Corresponding Secretary;*

" S. P. Harrington, *Treasurer.*

Drs. Riley and Harrington were appointed to conduct the President elect to the chair.

On taking the chair, the President, after thanking the members for the honor conferred, and asking their assistance in the discharge of the duties they had just imposed upon him as presiding officer, declared the Central Ohio Dental Association to be in working order, and exhorted the members to work constantly and laboriously for the accomplishment of its aims and interests.

On motion of Dr. Riley, Drs. W. E. Ide and J. W. Baker, of Columbus, were elected honorary members.

Dr. M'Cune moved an amendment to the Constitution, to the effect that honorary members be exonerated from assessments, which was laid over until the next meeting.

Dr. W. S. Sedgwick presented to the Society a fine specimen of an abnormal human stomach, showing the effects of impure metallic plates.

On motion, the thanks of the Society were tendered to Dr. Sedgwick for his donation.

On motion of Dr. M'Cune, the dentists of Newark were appointed a committee to make arrangements for the next meeting of the Society.

On motion, the thanks of the Society were tendered to Dr. Riley for the use of his office.

On motion, the Society adjourned to meet at Newark, O., on the 10th and 11th of July, 1861.

(Signed), J. B. Beauman, *Sec'y.*

